# NIPBL^+/−^ haploinsufficiency reveals a constellation of transcriptome disruptions in the pluripotent and cardiac states

**DOI:** 10.1038/s41598-018-19173-9

**Published:** 2018-01-18

**Authors:** Jason A. Mills, Pamela S Herrera, Maninder Kaur, Lanfranco Leo, Deborah McEldrew, Jesus A Tintos-Hernandez, Ramakrishnan Rajagopalan, Alyssa Gagne, Zhe Zhang, Xilma R. Ortiz-Gonzalez, Ian D. Krantz

**Affiliations:** 1 Division of Human Genetics, The Department of Pediatrics, The Children’s Hospital of Philadelphia, Philadelphia, Pennsylvania 19104 USA; 2 Kirby Center for Molecular Ophthalmology and Center for Advanced Retinal and Ocular Therapeutics (CAROT), Scheie Eye Institute, The University of Pennsylvania, Philadelphia, Pennsylvania 19104 USA; 3 Division of Neurology, Children’s Hospital of Philadelphia, Philadelphia, Pennsylvania 19104 USA; 4 Department of Neurology, Perelman School of Medicine, University of Pennsylvania, Philadelphia, Pennsylvania 19104 USA; 5 Division of Genomic Diagnostics, Department of Pathology and Laboratory Medicine, The Children’s Hospital of Philadelphia, Philadelphia, Pennsylvania 19104 USA; 6 Center for Biomedical Health Informatics (CBHi), The Children’s Hospital of Philadelphia, Philadelphia, Pennsylvania, 19014 USA; 7 Center for Cellular and Molecular Therapeutics, The Children’s Hospital of Philadelphia, Philadelphia, Pennsylvania, 19104 USA; 8 Department of Pathology and Laboratory Medicine, The Children’s Hospital of Philadelphia, University of Pennsylvania, Philadelphia, Pennsylvania 19104 USA; 9 The Perelman School of Medicine at The University of Pennsylvania, Philadelphia, Pennsylvania, 19104 USA; 10 Department of Pathology and Laboratory Medicine, The Children’s Hospital of Philadelphia, Philadelphia, Pennsylvania 19104 USA

## Abstract

Cornelia de Lange syndrome (CdLS) is a complex disorder with multiple structural and developmental defects caused by mutations in structural and regulatory proteins involved in the cohesin complex. NIPBL, a cohesin regulatory protein, has been identified as a critical protein responsible for the orchestration of transcriptomic regulatory networks necessary for embryonic development. Mutations in *NIPBL* are responsible for the majority of cases of CdLS. Through RNA-sequencing of human induced pluripotent stem cells and *in vitro*-derived cardiomyocytes, we identified hundreds of mRNAs, pseudogenes, and non-coding RNAs with altered expression in NIPBL^+/−^ patient-derived cells. We demonstrate that NIPBL haploinsufficiency leads to upregulation of gene sets identified in functions related to nucleosome, chromatin assembly, RNA modification and downregulation of Wnt signaling, cholesterol biosynthesis and vesicular transport in iPSC and cardiomyocytes. Mutations in *NIPBL* result in the dysregulation of many genes responsible for normal heart development likely resulting in the variety of structural cardiac defects observed in the CdLS population.

## Introduction

Cornelia de Lange syndrome (CdLS) is one of a family of disorders known as *cohesinopathies*, or more broadly, disorders of transcriptional regulation (DTRs). It is a rare, dominant, genetic multisystemic disorder caused by a disruption of the normal function and regulation of the cohesin complex; a key regulator of cell division and gene expression during development^1,2^. CdLS arises from heterozygous mutations in cohesin structural and regulatory proteins. There have been five causative genes identified in CdLS - *NIPBL, SMC1A, SMC3, RAD21, and HDAC8*^3–8^. About 60% of all diagnosed cases result from mutations in *NIPBL*, which is required for the loading and unloading of cohesin onto DNA^9,10^. It has been shown that as little as a 15% decrease in *NIPBL* expression can lead to multiorgan defects, including disruption of gut and heart development during embryogenesis^11–14^. Although cohesin’s canonical role in sister chromatid segregation is not disrupted in CdLS, the precise cellular and molecular mechanisms underlying morbidity or mortality still remain enigmatic. Several key biological and cellular features of this diagnosis have been documented, including altered transcriptional regulation, DNA replication, homologous recombination-mediated repair, genome compartmentalization, and RNA biogenesis^14–17^. Typical characteristics of children with CdLS may include clinical features of intellectual disability, growth retardation, facial dysmorphism, microcephaly, limb anomalies, hypertrichosis, and congenital heart defects.

Congenital heart disease (CHD) is the most common life-threatening birth defect in humans affecting approximately 1% of all newborns^18^. CHD is a significant cause of morbidity and mortality in children with CdLS, affecting 30–40% of patients^13,19^. Cardiac abnormalities vary across patients with most involving structural anomalies; atrial and ventricular septal defects and pulmonic stenosis tend to be most frequently diagnosed, with more severe, yet occasional, anomalies including Tetralogy of Fallot (TOF) and single ventricle malformations. To better investigate these congenital anomalies, several NIPBL deficient animal models have been created. NIPBL haploinsufficient mice manifest several features of CdLS and are significantly smaller than wildtype siblings and display structural heart defects^11,13,20^. Studies have shown that these animal models correlate with patient clinical phenotypes, one study found 77% of NIPBL^+/−^ mice had incomplete ventricular septation, compared to 14% of the wildtype mice^12^. New allelic series (NIPBL^FLEX^) models that allow for creation and efficient rescue of *NIPBL*-expression by targeting multiple lineages at early developmental stages suggest that multiple germ layers and cell lineages contribute to the CHD risk in CdLS patients^12^. Although this system has many advantages, the mouse models have obvious limitations. For example, the transgenic mice do not carry the same mutations as human patients. Although the conditional mutant phenotypes are observed in cardiac morphogenesis, these models are unable to validate if these defects occur in very early embryos. Recent advancements in pluripotent stem cell technology have overcome these limitations, and have allowed us to generate patient-specific strategies for studying the pathogenic mechanism of CdLS in multiple cell types and at the earliest stages of embryogenesis.

It has been hypothesized that the multi-organ defects commonly observed in CdLS patients are due to a global disruption of transcriptional regulation; which, due to an overall NIPBL deficiency, leads to altered epigenetic, biological and cellular responses. To access the impact of NIPBL on gene expression in human pluripotency and early cardiac development, we performed genome wide transcriptome analysis from pluripotent stem cells and cardiomyocytes from male and female NIPBL patient samples. Here, we report that hundreds of genes are differentially expressed genes at the iPSC and cardiomyocyte stage between control and NIPBL^+/−^patient generated samples and overlapping biological and cellular processes involve increased chromatin modifications, nucleosome assembly and transcriptional regulation, and reduced immunological functions, regulation of apoptosis and proteasome dynamics. Alteration in expression of many known CHD associated genes suggest that variability in CdLS birth defects results from a constellation of cardiac signaling events.

## Results

### Development of an *in vitro* iPSC cardiac model for CdLS

To investigate the impact of NIPBL mutations in CdLS patient samples, we focused our work on the role they play in cultured human cells, both in the undifferentiated and cardiomyocyte states. The role of NIPBL has been extensively studied in *Drosophila*^21–24^, murine^12,25,26^, zebrafish^13,20^ and human skin, blood and lymphoblastoid cell lines^14,27^. The cohesin-accessory protein NIPBL, has been previously implicated in regulating the pluripotent cell state and has been identified as a key component in defects of limb and cardiac development in NIPBL^+/−^ patients presenting with structural heart defects^2,25,28,29^; however, the mechanism by which it affects cardiac development has remained unclear.

**Figure 1:**
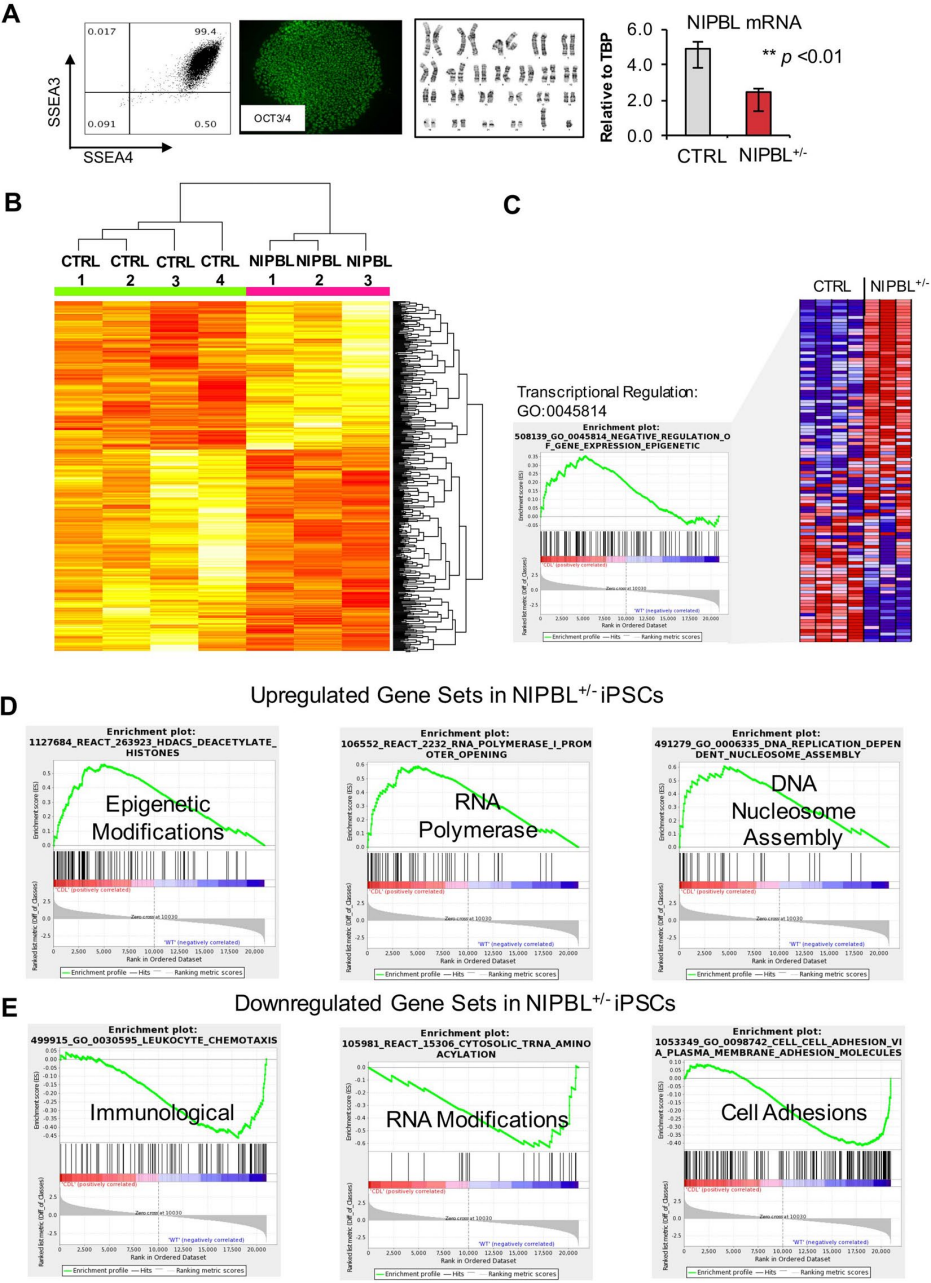
Cornelia de Lange transcriptome signature in patient derived iPSCs. (**A**) Representative characterization of NIPBL^+/−^ iPSCs, showing pluripotency surface marker and intracellular markers, a normal diploid karyotype, and reduced *NIPBL* expression in IPSCs. Full characterization in Supplemental Fig. 1. (**B**) Heat map of the top 183 and 250 DEGs with higher (red) and lower (yellow) expression in CDL. Each row represents a DEG, whose expression measurements are normalized across samples. Samples are clustered by these genes and the columns are colored (green = CTRL and pink = NIPBL^+/−^). (**C**) GO term analysis (GO-0045814) showing genes upregulated and downregulated in NIPBL^+/−^-iPSCs associated with transcriptional regulation. (**D,E**) Representation of upregulated gene sets enriched for NIPBL^+/−^-iPSCs and downregulated gene sets enriched for NIPBL^+/−^-iPSCs at FDR <0.5. Data from each individual was collected from two separate clones per replicated and a pool of RNA for the third technical replicates, and error bars are representative of ± SEM.

CdLS is caused by impaired function of the cohesin complex leading to numerous transcriptional and epigenetic regulation defects^10,16^. To generate a model to study transcriptional disturbances underlying the cardiac phenotypes seen in CdLS, we established iPSCs using episomal reprogramming methods^30,31^ (Fig. 1A, Supplemental Fig. 1A–F). Fibroblast and LCLs were obtained from four patients to generate iPSC representative of their CdLS phenotype, and each containing a recurrent NIPBL gene mutation (Supplemental Fig. 1A). Each established cell line showed reduced mRNA expression between 60–75% compared to unaffected controls (Supplemental Fig. 1B). We established upwards of three iPSC lines per subject and ≥2 of these cell lines were used for iPSC transcriptome analysis or cardiac differentiations. The generation of patient specific NIPBL^+/−^ iPSC lines did not present any problems, and displayed reprogramming efficiencies similar to that of control cell lines previously generated and specifically matched to each patient line^32,33^.

There were no noticeable morphological differences observed when culturing NIPBL^+/−^-iPSC and control-iPSCs. Haploinsufficient NIPBL cell lines possessed a high expression of pluripotent surface antigen expression (SSEA3 and SSEA4), and stained positively for intracellular pluripotency markers (OCT4 and Nanog); displaying similar levels of core pluripotency RNA gene expression by RT-PCR in comparison to human embryonic cells (H9) (Supplemental Fig. 1C,D). In addition, all cell lines studied were karyotypically normal over >20 passages and SNP arrays showed no evidence of CNVs (Supplemental Fig. 1E). Differentiation capacity was determined by *in vivo* germ layer teratoma assay, which was assessed through histological analysis which identified that all three germ layers (endoderm, ectoderm and mesoderm) were present (Supplemental Fig. 1F).

### NIPBL deficiency results in altered gene expression in the pluripotent state

Increasing evidence suggests that mutations in NIPBL impact the transcription of a number of genes throughout the genome, which can lead to alteration in the processing and assembly of key cellular complexes necessary for proper cell maintenance and differentiation. To investigate the extent of transcriptional deregulation in three NIPBL-mutation positive IPSCs, we carried out high-throughput global transcriptome RNA sequencing (RNASeq) and compared the gene expression profiles to four unaffected control subjects. All samples were quality controlled for pluripotency markers and genome stability prior to RNA-analysis, and NIPBL^+/−^-iPSC showed significant reduction in NIPBL RNA expression. It is worth noting that all RNA was collected from two separate clones and a pool of these clones for a total of three technical replicates per individual for all transcriptome analyses. The expression profiles of the NIPBL^+/−^-iPSCs were clearly distinguishable from the control subjects, and thus we performed differential expression analysis on our samples and identified that there were 183 upregulated and 250 downregulated genes in NIPBL^+/−^-iPSCs (*p < 0.05; Fig. 1B). When we focused on pluripotency associated genes using gene set enrichment (GO:0007507), we did not identify significance between unaffected control and NIPBL^+/−^-iPSCs; however, in concordance with previous molecular findings in CdLS, transcriptional regulatory genes were significantly enriched in NIPBL^+/−^-iPSCs (Fig. 1C). Additionally, further gene ontology analysis of all differentially expressed genes (DEGs) showed that upregulated and downregulated genes sets in NIPBL^+/−^-iPSCs belong to different categories in biological processes, cellular components, and molecular functions. Upregulated gene sets were associated with chromatin modifications (nucleosome assembly, chromatin silencing, DNA methylation on cystine), and RNA polymerase modification (polymerase promoter opening, elongation, transcription) (Fig. 1D), and downregulated gene sets included immunological functions (neutrophil migration and chemokines), RNA modifications, and cell-cell adhesion (Fig. 1E). Overall, NIPBL deficiency results in substantial transcriptional perturbations in patient derived NIPBL^+/−^-iPSC with the data showing hundreds of dysregulated genes.

### NIPBL^+/−^ cultured cardiomyocytes reveal widespread dysregulation of congenital heart disease (CHD) associated genes

**Figure 2:**
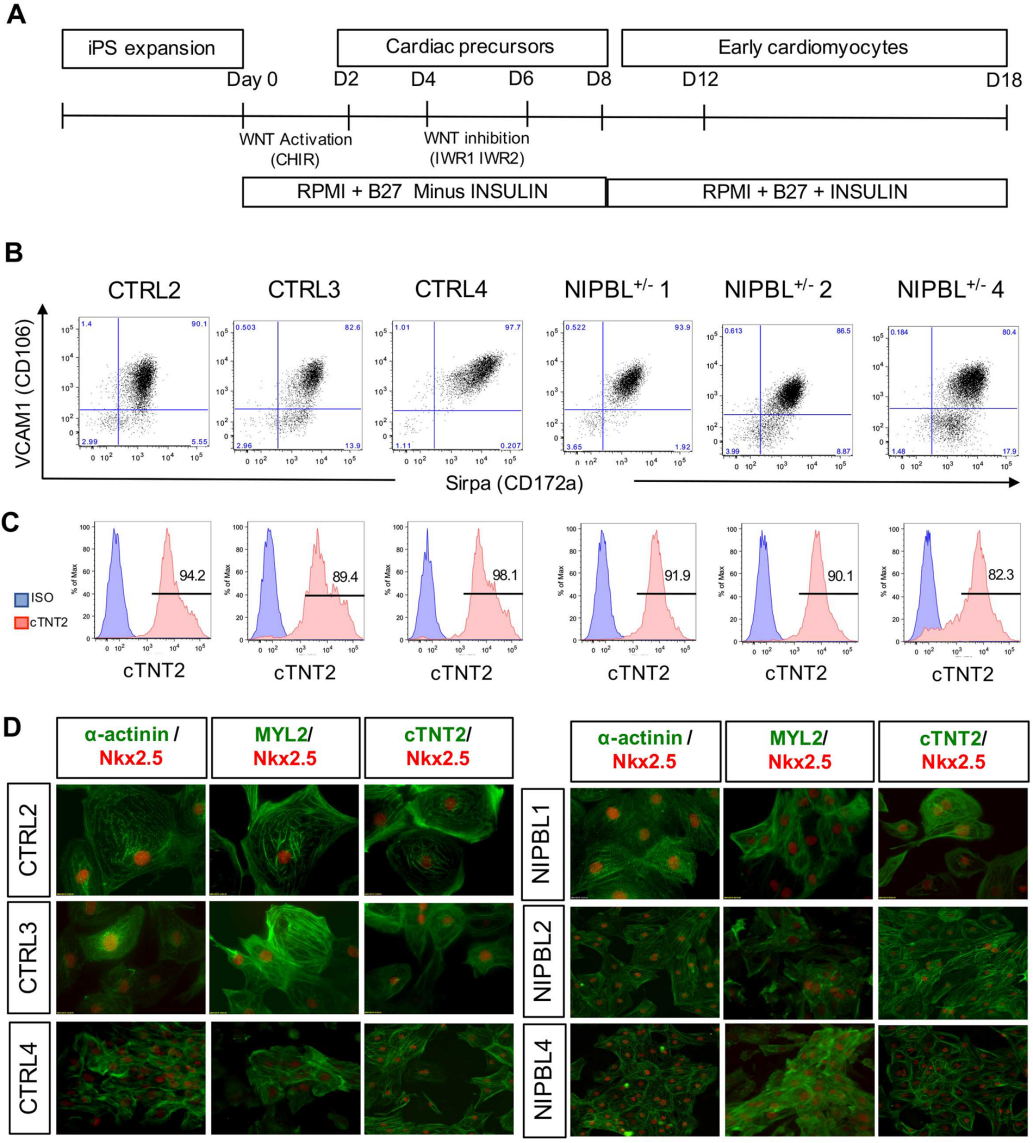
Characterization of cardiomyocytes from CTRL and NIPBL^+/−^ derived iPSCs cultures. (**A**) Schematic of cardiomyocyte differentiation from human iPSCs. (**B**) Flow cytometry analysis of day 18 cardiomyocytes derived from 3 CTRL and 3 NIPBL^+/−^-iPSCs for VCAM1 and SIRPA extracellular marker expression. (**C**) Intracellular flow cytometry analysis of cardiac troponin T (cTNT2) shows high enrichment of cardiac cells from CTRL and NIPBL^+/−^-iPSCs. (**D**) Immunofluorescence of cardiac specific proteins; alpha-actinin (A actinin), myosin light chain (MYL2), and cardiac troponin T (cTNT2), and nuclear staining of NKX2.5. Samples from each individual contained 3 technical replicates from ≥2 clones, and a minimum of 3 separate differentiations.

To gain further insight into how these changes may influence heart development, we investigated the capability of these patient derived cells to generate cardiomyocytes *in vitro*. CdLS patients exhibit multiple forms of congenital heart defects. In order to develop a model amenable for studying early stages of heart development, we investigated the capacity of NIPBL^+/−^-iPSCs to generate uniform populations of contracting cardiomyocytes. This was performed using a cardiac-directed monolayer differentiation through modulation of the WNT and insulin pathway at specific time intervals (Fig. 2A)^34^. The cardiac differentiation from iPSCs to early cardiomyocyte progenitors occurred over eight days, at which point beating cardiomyocytes were observed in both unaffected controls and NIPBL^+/−^ patient derived cell lines. When comparing culture growth rates between unaffected controls and NIPBL^+/−^ patient lines, no noticeable difference in cell numbers or proportion was observed. High population yields confirmed a successful lineage commitment to cardiomyocyte in both control and NIPBL^+/−^ patient lines, and thus a well-established human disease model for studying transcriptional variation in NIPBL^+/−^ cardiac specific cells.

**Figure 3:**
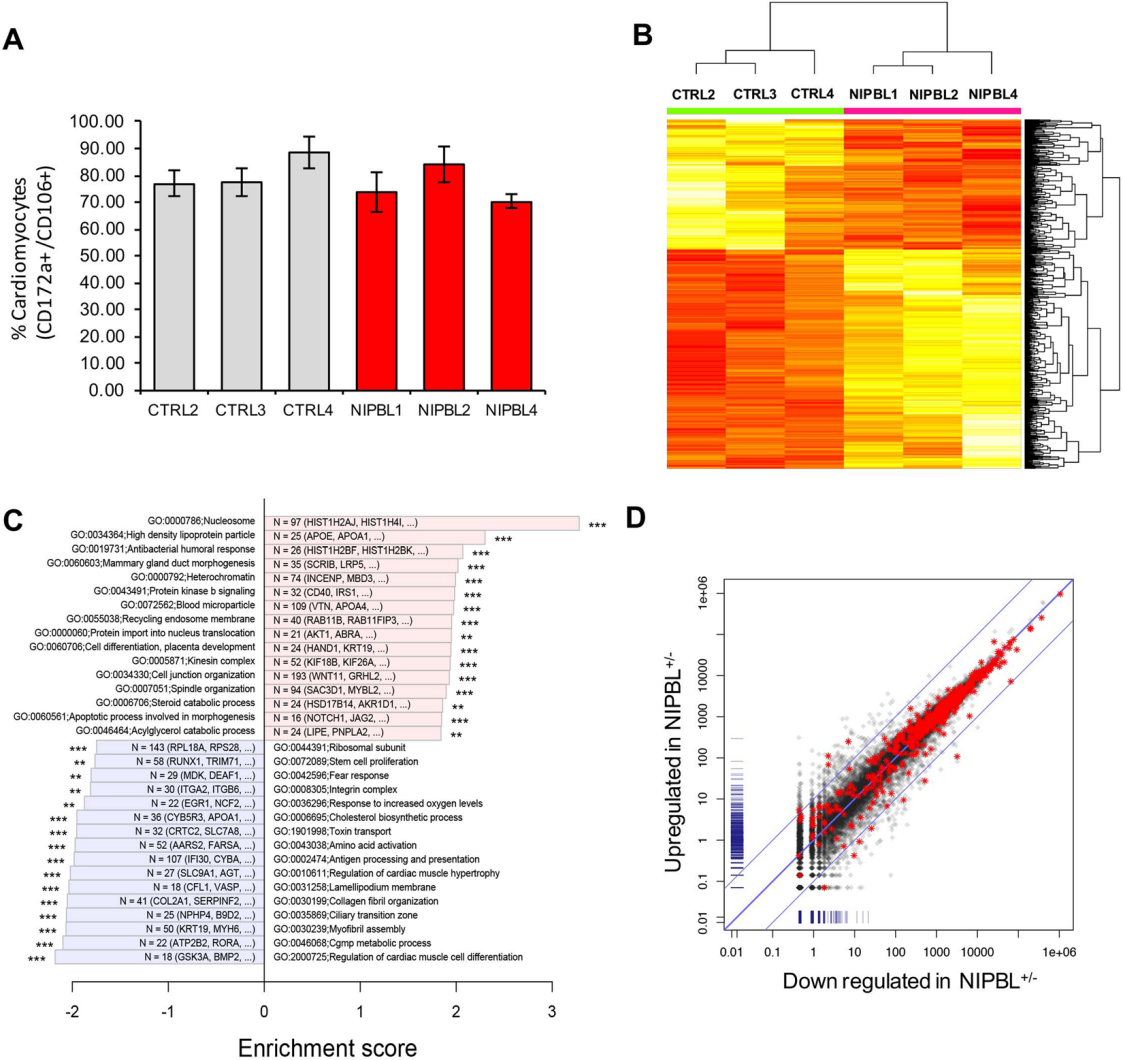
Identification of the effector gene sets responsible for NIPBL^+/−^ cardiomyocyte transcriptome dysregulation. (**A**) Quantification of cardiomyocytes generated from control and NIPBL^+/−^ iPSCs measured by surface marker expression (CD172a/CD106) (**B**) Heat map of the top 422 and 250 DEGs with higher (red) and lower (yellow) expression in NIPBL^+/−^ CM. Each row represents a DEG, whose expression measurements are normalized across samples. Samples are clustered by these genes and the columns are colored (green = CTRL and pink = NIPBL^+/−^). (**C**) Each bar represents a top GO term that was generally changed in NIBPL^+/−^ cardiomyocytes according to Gene Set Enrichment Analysis (GSEA). Go terms were selected based on significance and non-redundancy. Bar length is the nominal enrichment score of GSEA (positive = higher in NIBPL^+/−^). Labels show the total number of genes in each GO term and two top genes with the most significant change in NIBPL^+/−^. Significance of change was indicated by number of asterisks (**: less than 0.01 and ***: less than 0.001), (**D**) Genes related to heart development (GO:0007507) are highlighted in red. Among the total of 497 genes, 40 were significantly changed in patients (**p < 0.01). The x-axis represents the downregulated genes in and the y-axis are upregulated genes with a fold change >2 in the NIPBL^+/−^ CM compared to controls. Data from each individual was collected from two separate clones per replicated and a pool of RNA for the third technical replicates, and error bars are representative of ± SEM.

Once differentiated, cardiomyocytes were characterized through the quantification of cardiac specific marker expression. Flow cytometry was used to verify expression of cardiac extracellular surface markers; SIRPA + (CD172a) and VCAM1 + (CD106)^35^,which demonstrated little to no significant quantifiable difference between control and NIPBL^+/−^ cardiomyocyte cultures (Fig. 2B). Upon confirming extracellular expression of cardiac specification, immunostaining of intracellular proteins was performed using flow cytometry as well as immunofluorescence staining. Cardiomyocyte myofilament proteins were targeted, such as cytoskeletal actin-binding protein markers: alpha-actinin (α-actinin), myosin light chain protein (MYL2), and contractile marker troponin T (cTNT2); along with cardiomyocyte transcription regulator, NKX2.5 (Fig. 2C,D). Positive expression of these proteins confirmed the specification of control and NIPBL^+/−^ cardiac populations at nearly equal proportions, with control cardiomyocytes at 77–88% and NIPBL^+/−^ patient-lines at 70–84% purity (Fig. 3A). Although the data suggests there are no roadblocks in the generation of early NIPBL^+/−^ cardiomyocytes in our *in vitro* differentiation system, global gene analysis of unaffected control versus NIPBL^+/−^ cardiomyocyte cultures identified significant alterations in hundreds of genes (Fig. 3B).

RNA sequencing results identified 422 upregulated and 250 downregulated statistically significant DEGs in NIPBL^+/−^ cardiomyocytes when compared to unaffected controls (*p < 0.05). The complexity of gene regulation defects in NIPBL^+/−^ cardiomyocytes extends to many biological and cellular phenotypes. We identified a number of Gene Ontology (GO) terms enriched in cardiomyocytes (Fig. 3C, **p < 0.01). Among these, upregulation of several genes was detected with association to chromatin (nucleosome, DNA packaging, DNA bending), HDL particles, protein kinase B and vesicular transport (recycling endosome, protein import, kinesin complex). Downregulation of several genes sets were observed for regulation of cardiac muscle cell differentiation, ciliary transition zone, cholesterol biosynthesis and mitochondrial and cytoplasmic ribosomal subunits.

When specifically focused on the gene ontology associated with heart development (G0: 00075075), we identified 10 genes that were significantly altered in NIPBL^+/−^ cardiomyocytes at ≥ 2-fold (Fig. 3D and Table 1, *FDR* < 0.05). In addition, we identified a set of coronary heart disease (CHD)-associated genes consisting of several transcription factors, structural proteins, several receptors, and signaling molecules^36^. We found that twelve were significantly dysregulated at a *p*-value < 0.05, but extending the confidence level to *p*-value < 0.15 an additional 9 genes were identified to be altered in NIPBL^+/−^ cardiomyocytes. This showed that 21/53 CHD-associated genes were differentially expressed in our NIPBL^+/−^ patient-derived cardiomyocytes (Table 2). Patients with CdLS have numerous structural birth defects ranging from those least prevalent, such as Tetralogy of Fallot (ToF), hypertrophic cardiomyopathy, and valve dysplasia to heart anomalies most prevalent and commonly seen in affected patients, such as pulmonary stenosis, and septal defects, both atrial and ventricular. Amongst CHD-related genes associated with septal defects (A/V) and ToF, we identified critical cardiac genes (*GATA4/6, MYH6/7, MYH7, ACTN2, HAND2, TBX1/5, TDGF1*) to be significantly altered in our NIPBL^+/−^ cardiomyocytes.

**Table 1:** Heart development gene altered in NIPBL^+/−^ cardiomyocytes Heart (GO: 0007507, FDR <0.05).

Gene ID	Description	LogFC	*p*-value	FDR
*TDGF1*	teratocarcinoma-derived growth factor 1	1.738	8.56E-05	1.92E-02
*GRHL2*	grainyhead-like transcription factor 2	1.613	3.61E-04	4.95E-02
*MOSPD3*	motile sperm domain containing 3	1.355	5.15E-04	5.94E-02
*WNT11*	wingless-type MMTV integration site family member 11	1.333	1.40E-05	6.14E-03
*STRA6*	stimulated by retinoic acid 6	1.265	1.32E-04	2.54E-02
*ZFPM1*	zinc finger protein, FOG family member 1	1.262	2.43E-03	1.33E-01
*SALL1*	spalt-like transcription factor 1	1.221	3.91E-04	5.17E-02
*GREM1*	gremlin 1, DAN family BMP antagonist	−1.622	3.94E-04	5.19E-02
*COL3A1*	collagen, type III, alpha 1	−1.702	2.04E-04	3.37E-02
*COL11A1*	collagen, type XI, alpha 1	−1.953	2.19E-05	8.49E-03

**Table 2:** Congenital heart disease (CHD) associated genes and expression pattern in NIPBL^+/−^ cardiomyocytes.

GENE	PROTEIN NAME	PHENOTYPES	*p*-Value	Expression
**Transcriptional regulators**				
*ANKRD1*	Ankyrin repeat domain	TAPVR	0.067	down
*CITED2*	c-AMP responsive element-binding protein	ASD; VSD	0.690	up
*GATA4*	GATA4 transcription factor	ASD, PS, VSD, TOF, AVSD	0.064	up
*GATA6*	GATA6 transcription factor	ASD, TOF, PS, AVSD, PDA, OFT defects, VSD	0.068	down
*HAND2*	Helix-loop-helix transcription factor	TOF	0.580	up
*IRX4*	Iroquois homeobox 4	VSD	0.650	down
*MED13L*	Mediator complex subunit 13-like	TGA	0.360	up
*NKX2.5*	Homeobox containing transcription factor	ASD, VSD, TOF, CoA, TGA, DORV, IAA, OFT defects	0.210	up
*NKX2.6*	Homeobox containing transcription factor	PTA	0.003	up
*TBX1*	T-Box 1 transcription factor	TOF	0.380	up
*TBX20*	T-Box 20 transcription factor	ASD, VSD	0.120	up
*TBX5*	T-Box 5 transcription factor	AVSD, ASD, VSD	0.100	up
*TFAP2B*	Transcription factor AP-2 beta	PDA	0.200	up
*ZFPM2*	Friend of GATA	TOF	0.480	down
*ZIC3*	Zinc finger transcription factor	TGA, PS, DORV, TAPVR, ASD, HLH, VSD, Dextrocardia, L-R axis defects	0.110	up
**Receptors, ligands, and signaling**				
*ACVR2B*	Activin A receptor typeIIB	PS, DORV, TGA, dextrocardia	0.730	up
*ALDH1A2*	Aldehyde dehydrogenase 1, family member A2	TOF		
*ALK2*	BMP receptor	AVSD, *endocardial cushion defects*	0.200	down
*CRELD1*	Epidermal growth factor-related proteins	ASD; AVSD, *endocardial cushion defects*	0.440	up
*CRYPTIC*	Cryptic protein	TOF; TGA; AVSD; ASD; VSD; IAA; DORV	0.340	up
*FOXH1*	Forkhead activin signal transducer	TOF, TGA	0.052	up
*GDF1*	Growth differentiation factor-1	Heterotaxy, TOF, TGA, DORV	1.000	0
*GJA1*	Gap junction protein alpha 1, Connexin 43	ASD, TAPVR	0.059	down
*JAG1*	Jagged-1 ligand	PAS, TOF	0.200	down
*LEFTY2*	Left-right determination factor	TGA, AVSD, IAA, CoA, L-R axis defects, IVC defects	0.009	up
*NODAL*	Nodal homolog (TGF-beta superfamily)	TGA, PA, TOF, DORV, dextrocardia, IVC defect, TAPVR, AVSD	0.490	up
*NOTCH1*	NOTCH1 (Ligand of JAG1)	BAV, AS, CoA, *early valve calcification*	0.062	up
*PDGFRA*	Platelet-derived growth factor receptor alpha	TAPVR	0.120	down
*SMAD6*	MAD-related protein, member 6	BAV, CoA, AS	0.056	up
*TAB2*	TGF-beta activated kinase	OFT defects	0.950	up
*TDGF1*	Teratocarcinoma-derived growth factor	TOF, VSD	0.00005	up
*VEGFA*	Vascular endothelial growth factor A	CoA, OFT defects	0.680	up
*VEGFB*	Vascular endothelial growth factor B	CoA, OFT defects	0.410	up
*VEGFC*	Vascular endothelial growth factor C	CoA, OFT defects	0.680	down
**Structural Proteins**				
*ACTC1*	Alpha actin 1, cardiac muscle	ASD		
*BMPR2*	bone morphogenetic protein receptor type II	*cardiac septation defects associated with pulmonary hypertention*	0.780	up
*CALM2*	calmodulin 2 (phosphorylase kinase, delta)	Catecholaminergic polymorphic ventricular tachycardia (CPVT)	0.28	down
*DLK1*	Delta-like 1 homologue (Drosophila)	*VSD*	0.130	down
*ELN*	Elastin	SVAS, PAS, PS, AS	0.420	up
*MYBPC3*	Myosin binding protein C, cardiac	Hypertrophic Cardiomyopathy (HCM)	0.820	up
*MYH11*	Myosin heavy chain 11, smooth muscle	PDA, Aortic Aneurysm	0.460	up
*MYH6*	Alpha myosin heavy chain 6, cardiac muscle	ASD, TA, AS, PFO, TGA, *hypertrophic cardiomyopathy*	0.022	up
*MYH7*	Beta myosin heavy chain 7, cardiac muscle	Ebstein anomaly, ASD, NVM	0.074	down
*MYL2*	Myosin light chain 2	*Hypertrophic Cardiomyopathy*	0.004	down
*NPPB*	Natriuretic peptide B	*ASD, heart failure*	0.084	down
*PLA2G2A*	Phospholipase A2 group IIA	*ASD*	0.77	down
*PLN*	Phospholamban	Hypertrophic Cardiomyopathy (HCM), CHF	0.39	down
*SCN5*	Sodium channel voltage gated, type V alpha subunit	Long QT syndrome type 3 (LQT3)	0.260	down
*TMEM190*	Transmembrane protein 190	VSD	0.540	down
*TNNI3*	troponin I type 3 (cardiac)	Hypertrophic Cardiomyopathy (HCM)	0.250	up
*TNNT2*	troponin T type 2 (cardiac)	Hypertrophic Cardiomyopathy (HCM)	0.850	up
*TPM1*	Tropomyosin 1 (alpha)	Hypertrophic Cardiomyopathy (HCM)	0.0001	down
*XDH*	Xanthine dehydrogenase	Hypertension, Xanthinuria	0.280	down

### Transcriptome profiles in iPSCs and CMs capture convergence of CdLS signatures compared to other model systems

**Figure 4:**
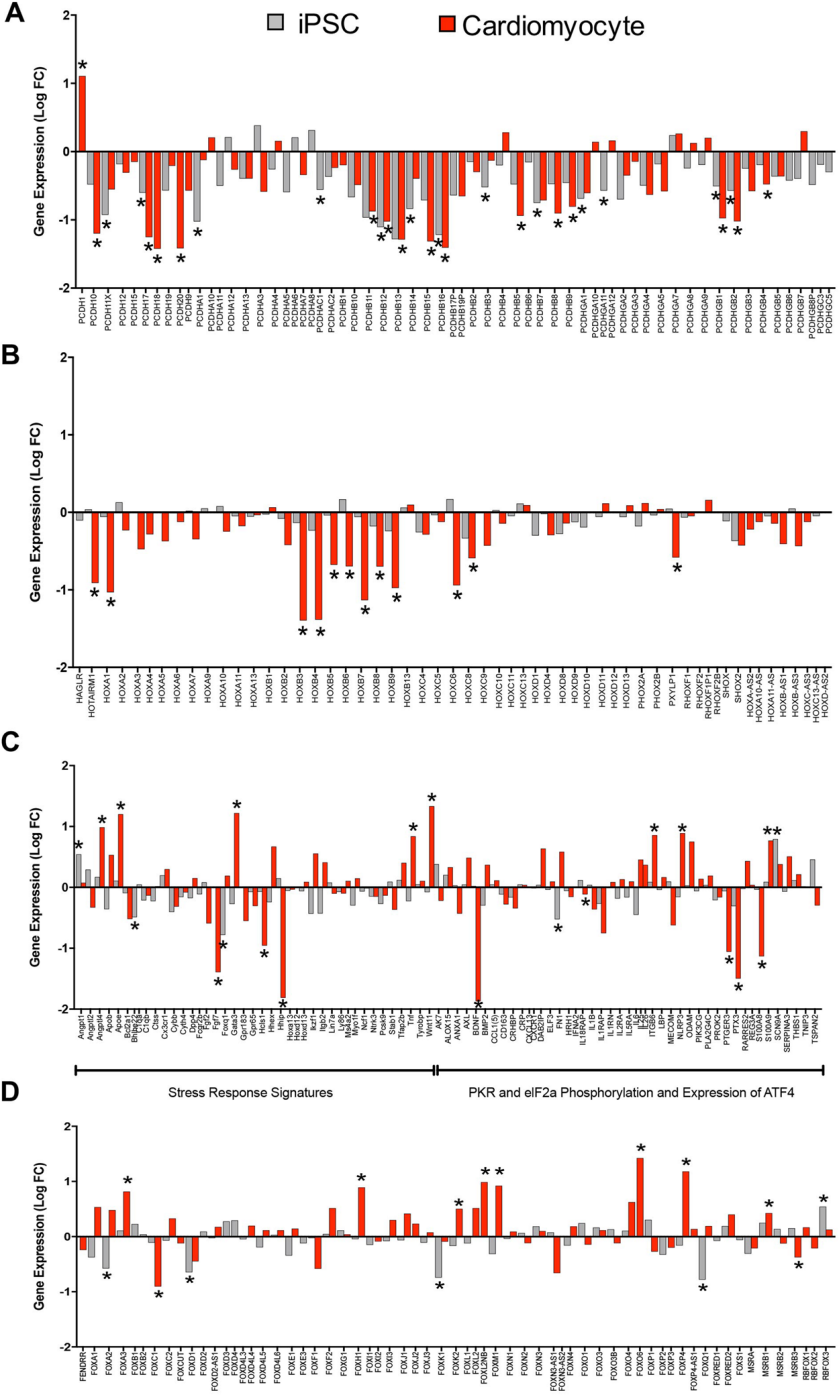
NIPBL^+/−^ suppresses expression of key developmental gene clusters. (**A,B**) mRNA analysis of Protocadherin (*Pcdh*) and _HOX_ gene clusters versus control iPSC and CMs. (**C**) mRNA analysis of PKR stress response pathway, divided into the stress response signatures and *PKR* and *elF2a* pathways genes. (**D**) *FOX* gene cluster gene expression analysis in NIPBL^+/−^ compared to controls. Data represents the average of gene expression for three NIPBL^+/−^ individuals (*p < 0.05, Data from each individual was collected from two separate clones per replicated and a pool of RNA for the third technical replicates, and error bars are representative of ± SEM.)

We next examined whether gene expression in NIPBL^+/−^-iPSC and -CMs correlated with previously identified gene set analysis in the literature. It was found that a set of transcriptome data generated from NIPBL patient-derived iPSCs and CMs correlated with murine data described by Remeseiro *et al*. for the protocadherin cluster (20 of 60 genes analyzed, Fig. 4A), which correlates with our gene set enrichment data for cell-cell adhesion dysregulation (GO_0098742). In contrast, HOX gene cluster genes show no differential gene expression differences in NIPBL^+/−^-iPSCs but NIPBL^+/−^CMs were identified to have 12 of 56 genes downregulated when compared to control-CMs (Fig. 4B). Notably, only 16 of the 86 genes previously shown to be associated with the stress responses pathways identified in fibroblast and LCLs from CdLS patient samples were correlated to our data in NIPBL^+/−^-iPSC or CMs (Fig. 4C)^17^. This possibly reflects cell-type-specific differences. Extending our comparisons to a gene cluster known to be active in cardiac development^37^, the forkhead box (*FOX*) gene cluster also showed significant dysregulation in either iPSC (5 of 64 genes) or CM (10 of 64 of genes) when compared to control samples but no genes overlapping in both cell types (Fig. 4D).

**Figure 5:**
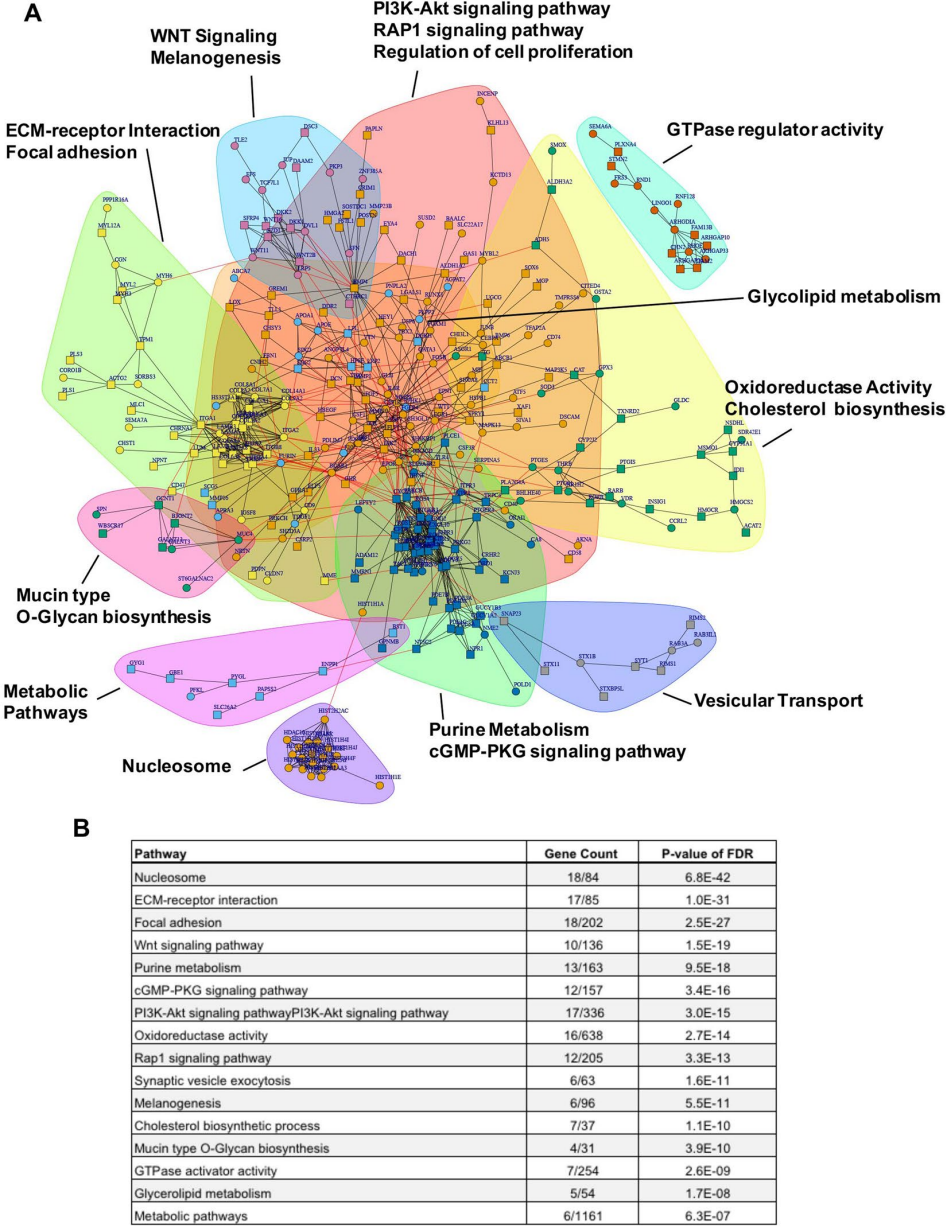
RNA-seq analysis of NIPBL^+/−^ iPSC and CMs (**A**) Gene interaction network analysis of differential gene expressed in both NIPBL^+/−^ iPSC and CMs. Top genes with higher (square) and lower (circle) expression in patients were connected to each other according to the STRING database of protein-protein interaction. Genes were grouped into 11 clusters by iGraph. Enrichment analysis of Goterms and KEGG pathways was applied to each cluster and all clusters combined. (**B**) Representative of GO enrichment for KEGG pathways listed with order of significance.

Our overarching goal was to find common networks associated with a biological or cellular function to better understand the role of NIPBL in embryonic and cardiac development. Our bioinformatics analysis showed an over-representation of 329 and 645 genes, either upregulated or downregulated (*p < 0.05), respectively in both NIPBL^+/−^ cell types. We used String database to determine the overlapping pathways enriched in our NIPBL samples and showed that the nucleosome is primarily affected, followed by ECM-receptor interaction, focal adhesion, Wnt signaling, purine metabolism, cGMP-PKF, and PI3K-Akt signaling pathways were affected to the greatest degree (Fig. 5A,B). RNASeq results for overlapping pathways and cardiac CHD-associated gene expression were validated by qRT-PCR for 10 genes. Data showed similar pattern of expression for epigenetics modifiers, ECM-interactors and adhesion molecules, and CHD-genes as those generated through RNASeq analysis (Supplemental Fig. 2). Taken together, this data confirms and expands the catalogue of gene and pathways currently identified to be regulated by the cohesin complex (primarily or downstream) as a result of NIPBL haploinsufficiency in iPSC and CMs.

## Discussion

CdLS is a genetically heterogeneous disorder with multiple structural and developmental deficits. Elucidating the mechanisms by which disruption of cohesin function results in these pleiotropic manifestations is challenging. A number of non-human models have been developed for *in vivo* studies, but none recapitulates the human disease completely, underscoring the need for the development of human CdLS-related disease models. To investigate the consequence of NIPBL haploinsufficiency in early embryonic and cardiac development, we generated induced pluripotent stem cells from NIPBL^+/−^ patients and differentiated these cell lines to cardiomyocytes. The results of this investigation complement previous studies suggesting that CdLS is a disorder of global transcriptional disturbances in human, drosophila, murine and zebrafish samples^12–15,17,20–22,26^. The reduced expression of NIPBL (60–75% of normal) resulted in no difference in reprogramming efficiency, loss of genomic stability, or alterations in cell morphology, pluripotency markers or proliferation. This is in contrast to Kagey *et al*. (2010), which showed that reduction of *NIPBL* using shRNA resulted in loss of pluripotent stem cells morphology, and reduction of key pluripotency mRNAs (*Oct3/4*, *SOX2*, and *Nanog*). Patient samples carrying mutations in *NIPBL* do not have a loss of mRNA expression greater than 40% of normal suggesting a threshold of expression necessary for maintaining cellular embryonic and somatic survival. Thus, using shRNA to knockdown *NIPBL* expression could have resulted in levels (<60%) which was not compatible with maintaining normal pluripotent stem cells characteristics. Although there were similarities in pluripotency characteristics in NIPBL^+/−^ compared to controls, iPSC transcriptome profiles show an array of gene disturbances similar to that observed in *NIPBL* shRNA knockdown studies. Our group and others have previously shown global transcriptional differences in CdLS samples; human fibroblast and lymphoblastoid cell lines, drosophila brains, murine and zebrafish tissues highlighting why it has been termed a “transcriptomopathy” and a member of a growing list of disorders of transcriptional regulation (DTRs)^38^. Given the strong involvement of transcriptional regulation and epigenetic factors in CdLS it was not surprising that we identified gene set alterations involving gene expression regulation, RNA polymerase, RNA modification, chromatin and that nucleosome-related genes were amongst the top hits in our bioinformatic analysis of NIPBL^+/−^-iPSCs.

The development of congenital heart defects involve perturbations of pathways responsible for cardiac morphogenesis, nutrient metabolism, myocyte specification, and neural crest and germ layer differentiation (CHDs)^36^. It is estimated that 25% of CdLS patients have some form of CHD but the etiology remains unclear; however, it has been suggested that small changes in many genes results in the wide variation of structural birth defects^11,14,19,20,39^. When we performed gene set enrichment analysis for heart development (GO: 00007507), 10 genes were identified to be dysregulated at >2-fold change, and FDR <0.05, but more than 80 genes with a *p*-value of <0.05 did not make the cut off for 2-fold change. The wide-range of phenotypic variability in *NIPBL* haploinsufficient patients is documented and can have CHDs ranging from significant structural heart defects to minor findings including innocent murmurs and persistent fetal circulatory issue^19^. We investigated the expression of known CHD-related genes to identify if one or a series of genes or pathways was significantly perturbed in NIPBL^+/−^ cardiomyocytes. Overall, genes involved in transcriptional regulation (*ANKRD1, GATA4, GATA6, NKX2.6*), receptor signaling (*FOXH1, GJA1, LEFTY2, NOTCH1, SMAD6, TDGF1*), and structural proteins (*MYH6, MYH7, MYL2, NPPB, TPM1*) were found to be consistently dysregulated in our cellular assays. Our results suggest that the CHDs seen in individuals with CdLS caused by *NIPBL* mutations are likely not a result of disruption in any one cardiac gene or developmental pathway, but rather result from a constellation of multiple gene/pathway dysregulation that in turn manifests in the broad spectrum of structural CHDs observed in CdLS patients. Although not studied here, these transcritptional dysregulations are likely additionally influenced by individual patient genetic background and environmental modifiers. Various CdLS models have been studied to define patterns of transcriptional dysregulation, which correlated with our findings in this study. Some of the greatest changes in expression occur within large gene clusters responsible for developmental gene regulation, such as the *HOX*, *Pcdh*, and *FOX* gene clusters^25,40–43^. While many promoters are unaffected by cohesin disruption, certain promoter regions such as those for the protocadherins and *HOX* gene clusters have been shown to be positively regulated by cohesin loading^10,44^. Consistent with these findings, more than 30% of *Pcdh* genes are significantly downregulated in both iPSCs and CMs. Regulation of *Pcdh* and *HOX* expression is hampered in *NIPBL* mutant murine and zebrafish models, and the underlying mechanism has been suggested to be dependent of the positional effects of the given *Pchd* or *HOX* gene within the clusters. The transcriptional activity of the clustered *Pcdhs* has been directly correlated with binding of CTCF/cohesin complex to active promoters and enhancers^45^, and regulation of expression is mediated through DNA-looping interactions within the Pcdh locus^41^. Many of the differences observed between *Pcdh, HOX*, and *FOX* genes could be associated with differences in embryonic developmental states and chromatin condensation effects during cell differentiation processes which are affected in both the iPSCs and CMs of NIPBL^+/−^ patient derived samples^25,28,44,46^. Our data shows significant disturbances in chromatin associated complexes (nucleosome and DNA bending), and modifiers of gene expression through epigenetic modification (*HDAC*, silencing of expression, and methylations). Interestingly, we find that 17% of the genes identified by Yuen *et al*. (2016) for the PKR stress response pathway in lymphoblastoid cell lines (LCLs) to be correlated with NIPBL^+/−^-iPSC and CMs.

Using an unbiased evaluation of RNA sequencing data, we identified unique disease-specific protein interaction networks overlapping in NIPBL^+/−^-iPSCs and cardiomyocytes. The most significant GO enrichment was related to genes of the nucleosome. These data show significant increased expression of histone coding genes and inhibition of methylation specific modifiers, which support the role of NIPBL in maintaining DNA methylation and gene silencing status and chromatin compaction and architecture^6,47,48^. We identified ECM-receptor interaction and focal adhesion defects which are important in intracellular signaling and cell-adhesion kinases responsible for cell survival and cell specification^45,49^. We also identified Wnt signaling alterations, which has broad implication for embryonic development and also substantial impact on cardiovascular development including cardiomyocyte polarity and left-right patterning^12,20,47,50^. Pistocchi *et al*.^51^ showed a reduction in canonical Wnt pathway with a subsequent decrease in *Ccnd1* expression in a nipblb-loss-of-function zebrafish model, which correlates with our iPSC and CM data which show altered Wnt signaling and significant reduction in *Ccnd2* expression and slight reduction in *Ccnd1* levels (not significant) in *NIPBL* patient samples. Dysregulation in Wnt signaling caused cardiac neural crest development defects in *Rad21*-depleted animals^52^. Interestingly, *NIPBL* mutations decreased the expression of cholesterol biosynthesis and glycolipid metabolism genes. Cholesterol and lipid metabolism is essential for embryonic development, abundant in the cardiovascular and central nervous system, and critical for plasma membrane components and signal pathways^53–55^.

In summary, our work has demonstrated that NIPBL^+/−^-iPSC and cardiomyocytes reveal considerable global transcriptional variations as compared to unaffected control cell lines. Perturbation of multiple biological, molecular and cellular processes highlight the complexity of primary and downstream effects mediated by dysregulation of the cohesin complex. This suggests that global alterations in gene expression during early cardiac development in CdLS is not directly related to a “monogenic” associated alteration in cardiac signaling, but rather consists of a cascade of effector genes which are regulated by epigenetic and DNA looping related alterations responsible for the phenotypic variations seen in CdLS-related structural heart defects.

## Experimental Procedures

### Human induced pluripotent stem cells generation from NIPBL probands

Primary skin cells were collected from four Cornelia de Lange (CdLS) probands carrying heterozygous mutations in the *NIPBL* gene (2969delG, 2479_2820delAG, 3503_2A > C, 2479_2480delAG; NIPBL1, NIPBL2, NIPBL3, NIPBL4, respectively) and all human protocols for this work were approved by the human subject Research Internal Review Board (IRB 98-001439) at The Children’s Hospital of Philadelphia. The methods performed in these studies followed all relavent guidelines and regulations set forth by institutional guidelines. Informed consent was obtained from all subjects involved in this work. Unaffected control induced pluripotent stem cells were generated as described previously^30,32,33,56^. iPSC-like colonies were expanded on MEFs in HES media containing 5 ng/mL bFGF, further characterization included quantitative polymerase chain reaction (PCR), immunofluorescence, flow cytometry for pluripotency marker expression and teratoma analysis. Additional details are provided in supplementary methods.

### Cardiomyocyte (CM) differentiation

iPSCs were cultured on 0.1% gelatinized plates containing iMEFs in 37 C 5%O_2_ and 5% CO_2_. Cell lines were feeder depleted twice and seeded onto matrigel-coated dishes. Cells were maintained until approximately 60% confluence in HES media (50%) and iMEF-conditioned media that was collected 36 h after plating iMEFs. The second feeder depletion occurred between 2-3 days prior to differentiation to completely eliminate residual iMEFs. iPSCs were dissociated into single cells using Accutase (Life Technologies) and plated onto matrigel-coated dishes (BD, hESC-qualified matrigel) in HES:MEF-conditioned (50:50) medium +20 ng/mL of bFGF supplemented with Rho-kinase inhibitor (10 μM). Next day, iPSCs were fed with mTesr media (Stem Cell Technologies) and maintained to reach 100% confluency. On the first day, IPS cells were changed to cardiac induction media (CM1; RPMI-1640, 1 × P/S, 1XGlutamime, 2XB27 supplement minus insulin) containing 12 μM CHIR (Selleck). Cells were maintained in cardiac media for 24 hours in a 37 C 5%O_2_ and 5%CO_2_ incubator, then switched to cardiac base media for 48 h. On day 4, media was changed to CM1 containing WNT inhibitors (IWR1 and IWP2, Sigma-Aldrich). Cells were placed in a 37 C 5% CO_2_ incubator for the remainder of cardiac differentiation. On day 6, media was replenished with CM1 without WNT inhibitors until day 8. On day 8, culture media was replenished with cardiac media (CM2; RPMI-1640, 1XP/S, 1 × Glutamine, 2XB27 supplement containing insulin). Contracting culture generally occurred between day 8-10 of differentiation. On day 12, cardiomyocytes were expanded using 0.2% collagenase II (Worthington Biochemical Corporation) plus DnaseI for 20 minutes in 37 C 5% CO_2_ incubator, cultures were washed with 1XPBS to removed floating cells and then treated for an additional 20-min with 0.2% collagenase to detach cardiomyocytes. Cardiomyocytes were less resistant to early detachment then other non-cardiomyocyte cells. This step was used to enrich cardiomyocyte cells at early passaging time points. Cells were replated at high density (1 × 10^6^ cells/well of 6-well) in CM2 media containing 10 uM Rock inhibitor (Y27632, Tocris) and culture media changed 16 h post-plating with CM2 media only. This high density ensured equal cell number and contracting cultures across cell line for future analysis. Cells were maintained until day 18 then harvested for transcriptome analysis.

### Flow cytometry analysis of cardiomyocytes

Culture of cardiomyocytes were collected using accutase to ensure single cell suspension. A portion of cells were incubated with antibodies anti-CD107a (Biolegend VCAM1,1:200) and anti-CD172a (Biolegend Sirpa, 1:400) in PBS supplemented with 0.5%BSA + 0.1% Na Azide (FACS buffer) and incubated at RT for 15 min as previously described^35^. For intracellular staining, cells were fixed using BD Perm/Fix for 20-min at 4 C, washed 2 × with FACS buffer. Cells were incubated in either anti-mouse IGG1 isotype control or anti-cTNT2 (ThermoFisher; 1:400) for 30-min at RT. Goat anti-mouse-488 (ThermFisher; 1:500) antibodies were added per 5E5 cells and incubated at RT for 30-min before washing with FACS buffer. We analyzed stained cells on a BD FACS Aria and data analyzed using FlowJo Version 10.0.8 software (TreeStar).

### Bioinformatics

RNA-seq reads saved in.fastq files were aligned to human reference genome and transcriptome using the Spliced Transcripts Alignment to a Reference, or STAR, program (version 2.5.1b). The NCBI GRCh38 reference genome and transcriptome were downloaded from the iGenomes (https://support.illumina.com). STAR was run in its 2-pass mode: the first pass aligned reads to the reference genome and transcriptome using known splice junctions while allowing for detection of novel junction sites. Junction sites detected from all libraries by the alignment were collected, combined and filtered. The second pass re-aligned reads to the references using both known and novel junction sites. Aligned reads were mapped to known genes and reads with both ends uniquely mapped to the sense strand of the same gene were assigned to that genes. The mapping procedure generated a data matrix of read counts. Sample analysis using the matrix did not identify any batch effect or outlier sample. DESeq. 2 method was used to test for differential gene expression between WT and CdLS groups. GSEA (http://software.broadinstitute.org/gsea) method was used to identify gene sets enriched with differential expression. Top differentially expressed coding genes were clusterd by the iGraph (http://igraph.org) algorithm using the protein-protein interaction information downloaded from the STRING (https://string-db.org) database. The RNA- seq data is available through the GEO database library with the access ID: GSE102873. RNA was collected from two separate clones and a pool of these clones for a total of three technical replicates per individual for all transcriptome analyses

### Statistical Analysis

Quantitative data were obtained from three independent experiments per cell line (≥2). Statistical analysis was performed with Student T-test in Excel. *p*-values of ≤0.05 were considered statistically significant.

## Electronic supplementary material

Supplementary Experimental Procedure and Figures

Supplementary Dataset
