# Supplementary material for: NIPBL+/− haploinsufficiency reveals a constellation of transcriptome disruptions in the pluripotent and cardiac states
Source: Sci Rep. 2018 Jan 18;8:1056. doi: 10.1038/s41598-018-19173-9 (PMC5773608; doi:10.1038/s41598-018-19173-9)
Supplement: Supplementary file 1 — Supplementary Experimental Procedure and Figures [file 41598_2018_19173_MOESM1_ESM.pdf]

NIPBL$^{+/-}$ haploinsufficiency reveals a constellation of transcriptome disruptions in the pluripotent and cardiac states

Jason A. Mills[1,2]*, Pamela S Herrera[1,2], Maninder Kaur[1], Lanfranco Leo[2], Deborah McEldrew[1], Jesus A Tintos-Hernandez[3], Ramakrishnan Rajagopalan[4,5], Alyssa Gagne[6], Zhe Zhang[5], Xilma R. Ortiz-Gonzalez[3], Ian D. Krantz[1,7]*.

Supplemental Procedures

Human induced pluripotent stem cells generation from NIPBL probands

Fibroblast cell lines were expanded in Amniomax (Invitrogen), plus penicillin/streptomycin. Lentiviral or episomal reprogramming vectors were used to generate iPSC from probands as described previously for fibroblast cell lines[1-3]. The episomal vectors used for the NIPBL2, NIPBL3, and NIPBL4 fibroblast cell lines encoded *OCT3/4*, shRNA*p53*, *KLF4*, *SOX2*, *L-Myc*, *Lin28*, and *GFP* cDNAs. Briefly, one million cells per individual were harvested, and washed once with 1XPBS, and then 1ug/episomal plasmid (4ug total) were transfected using the Amaxa nucleofector (P-23 program). Cells were then placed into two 100-mm dishes containing MEFs, and maintained in HES media + 10ng/mL of bFGF for 2 days. Culture media was changed and cell were place into a new well in HES media + 10ng/mL of bFGF + 2uM sodium butyrate (NaB) for 7 days with media changes every other day. Cells were then sorted for all transfected cells containing *GFP*, and replated at 5000 cells/100-mm dish containing matrigel MEFs. Cells were maintained on dish for 14-16 days (day 21-23 post-transfection) where visible colonies were present with typical iPSC morphology. iPSCs were maintained in DMEM/F12 (50:50; corning) containing glutamine,

penicillin/steptomyocin, 15% Knockout serum replacement (KSR), 1X NEAA, 0.1mM β-mercaptoethanol (2-ME), and 5ng/mL of bFGF (R&D Systems) on 0.1% gelatin coated dishes with irradiated mouse embryonic fibroblast (iMEFs). Cells were maintained in 37C 5%O_2, 5% CO_2. Cell lines were expanded and characterized as previously described for morphology, pluripotency gene expression, surface marker expression[3,4].

Single Polymorphism (SNP) Microarray Analysis

DNA was isolated from each fibroblast and iPSC line using DNeasy Blood and Tissue Kit (Qiagen, Inc), and genotyped on the Illumina BeadChip at the Genomics Diagnostic laboratory, Children's Hospital of Philadelphia. The NIPBL1, NIPBL2, NIPBL3, and NIPBL4 fibroblast and iPSC lines were genotyped on HumanOmni1-Quad arrays with 1,140,419 markers. CNV analysis was performed using the PennCNV software5 based on the signal intensity (Log R Ratio and B Allele Frequency) data exported from Illumina GenomeStudio. The default parameters were used, and only large CNVs (>200kb) were retained for subsequent analysis. The signal intensity data around all CNV calls were plotted and visually inspected to ensure accuracy. We used the scan_region.pl program within PennCNV to determine if a CNV call is unique to iPSC lines, in comparison to the parental fibroblast as a reference.

Immunofluorescence

Glass coverslips are cleaned, autoclaved, and placed in the bottom of a 12-well tissue culture dish. Coverslips are then covered with 1:100 matrigel and placed in 37°C incubator overnight. The next day media is removed and dishes with matrigel + coverslips are placed at RT for 1h. Cells were plated onto coverslip dishes by dissociating to single

cells with Collagenase II (Worthington Biochemical Corporation), centrifuge cell suspension plus RPMI (Invitrogen) for 3 minutes. Resuspend pellet in cardiac media with rock inhibitor and plate on 12-well dish with coverslips. Maintain cells for 2-3 days or until a confluency of 30-40% has been reached and then prepare for fixation. Wash cells once with 1X dPBS. Fix cells by adding 4% paraformaldehyde in dPBS to each well, and let sit for 15 minutes at room temperature. Wash twice with cold dPBS for 5 minutes each and begin immunostaining of cardiomyocyte samples with cardiac specific antibody markers. A combination of Nkx2.5 (Abcam, Rabbit; 1:200) or alpha-actinin (Sigma iGG1; 1:100), cTNT2 (ThermoFisher; mouse iGG1; 1:300), MYL2 (Proteintech Group, Inc. mouse; IGG1; 1:200), MLCA (Synaptic Systems rabbit: 1:200) in immunofluorescence buffer (PBS, 1% goat serum, 3% BSA) was added to assigned wells and staining was performed overnight at 4°C. Next day, wash wells 1X with dPBS and stain with corresponding secondary antibody and let sit in dark for two hours, washed and counterstained with Hoechst or DAPI (DNA stain) for 10 minutes. Coverslips were mounted with ProLong Gold mounting medium (ThermoFisher Scientific). Slides were placed upright in slide box and stored in dark at RT to dry.

RNA isolation, library preparation and sequencing

Total RNA purification was carried out on iPSC and CMs as directed in PureLink^{TM} RNA Micro Kit protocol (Invitrogen) using QIAzol Lysis Reagent (Qiagen) including the optional on-column DNAse step. All samples were submitted for bioanalyzer analysis for quality control. Only samples receiving a RNA integrity number (RIN) score ≥9.4 were provided further RNA sequencing analysis. RNA-seq libraries were prepared with Illumina TruSeq

RiboZero Sample Prep kits according to the manufacturer's protocol and sequenced on a HiSeq 2500 (Illumina), using the paired end protocol with 100-bp read length to obtain three technical sequencing replicates per sample, each individual was prepared in triplicate.

First Strand cDNA Synthesis

RNA was isolated from iPSC or CMs and first strand synthesis was performed using TaqMan® Reverse Transcription Reagents. Protocol was followed as instructed by manufacturer, with each sample yielding a 20µL reaction from 1µg of RNA by using random hexamers as the primer type. cDNA was stored at -20C until needed for downstream analysis.

qRT-PCR analysis

Gene expression was quantified using the QX100™ Droplet Digital™ PCR system. Expression levels from all samples were measured using primer/probes to *NIPBL*, and normalized to *TBP* (ABI: 4325803). The ddPCR assay was performed as previously published[5]. Briefly, a 25uL reaction contained 20uL of ddPCR mastermix, 2.5uL cDNA (25ng cDNA) and 1.25uL of Target and 1.25uL of TBP primer/probe. Probes for targets contained FAM reporter and *TBP* genes contained VIC. Thermocycler conditions were 95C for 10 min, 95C for 15 sec then 58C for 30 sec for 35 cycle. For all experiments, a negative control (nuclease free H_20) was included alongside each sample. The expression levels for each sample were averaged across triplicate samples. For RNASeq validation, gene expression was performed using TaqMan Fast Advanced Master Mix

(Thermofisher, 4444557). qRT-PCR reactions contained 5uL of PCR mastermix, 2.5uL cDNA (12.5ng/uL), 1.25uL of Target and 1.25uL of *TBP* primer/probe. RNA sequencing validation was performed on genes: *HIST1H1A, HIST1H3A, HMGN5, PCDHB13, PCDHB16, CLDN4, CLDN19, GATA6, GATA4,* and *TDGF1*. Primer/probe information is provided in Supplementary Table 2.

SUPPLEMENTAL REFERENCE:

1. Okita, K. *et al.* A more efficient method to generate integration-free human iPS cells. *Nat Meth* **8,** 409–412 (2011).
2. Hubbard, J. J. *et al.* Efficient iPS Cell Generation from Blood Using Episomes and HDAC Inhibitors. *JoVE* 1–5 (2014). doi:10.3791/52009
3. Mills, J. A. *et al.* Clonal genetic and hematopoietic heterogeneity among human-induced pluripotent stem cell lines. *Blood* **122,** 2047–2051 (2013).
4. Sullivan, S. K. *et al.* High-level transgene expression in induced pluripotent stem cell-derived megakaryocytes: correction of Glanzmann thrombasthenia. *Blood* **123,** 753–757 (2014).
5. Kaur, M. *et al.* NIPBLexpression levels in CdLS probands as a predictor of mutation type and phenotypic severity. *Am. J. Med. Genet.* **172,** 163–170 (2016).

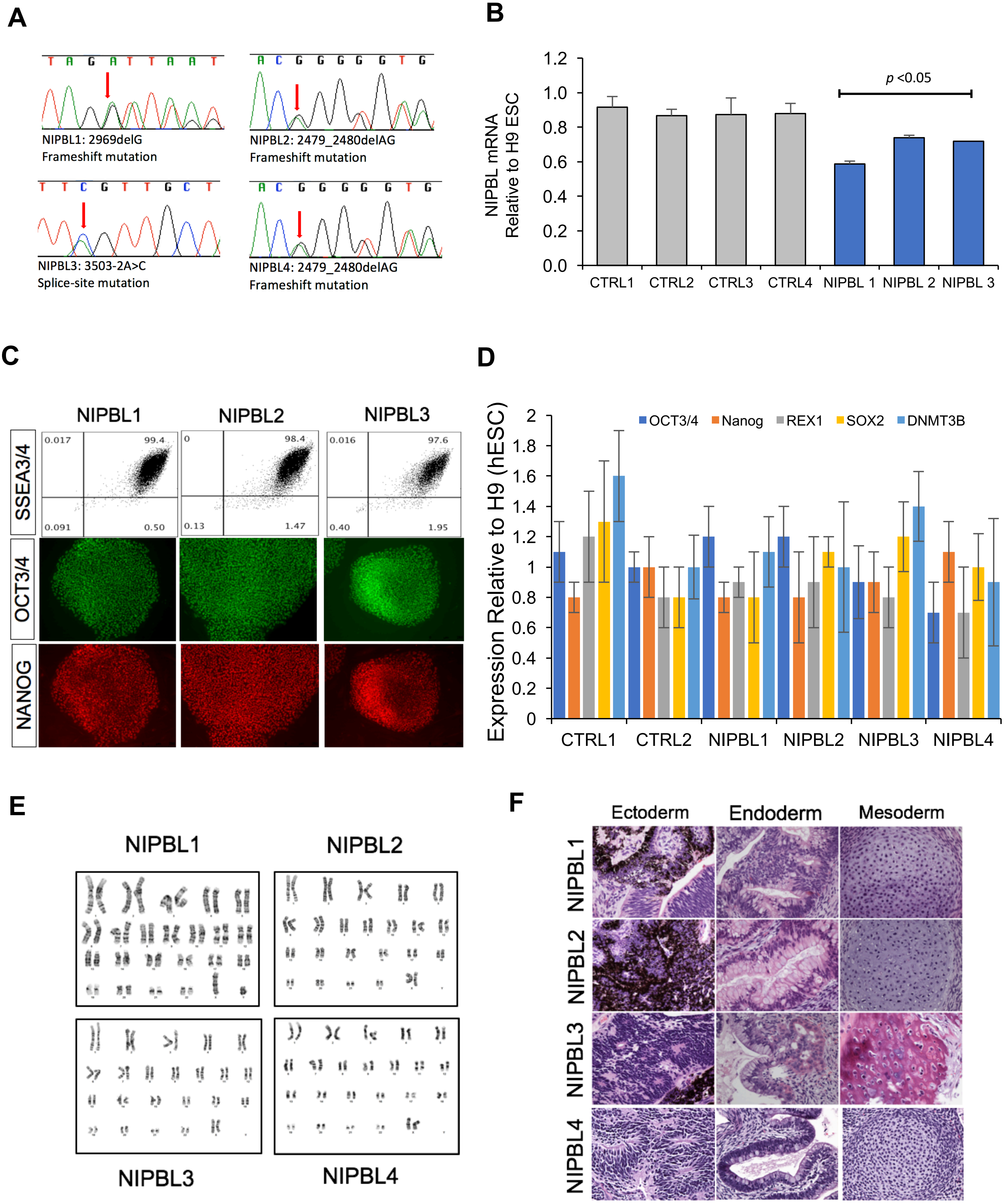
A
NIPBL1: 2969delG
Frameshift mutation
NIPBL2: 2479_2480delAG
Frameshift mutation
NIPBL3: 3503-2A>C
Splice-site mutation
NIPBL4: 2479_2480delAG
Frameshift mutation
B
NIPBL mRNA Relative to H9 ESC
p <0.05
CTRL1 CTRL2 CTRL3 CTRL4 NIPBL 1 NIPBL 2 NIPBL 3
C
NIPBL1 NIPBL2 NIPBL3
SSEA3/4
OCT3/4
NANOG
0.017 99.4 0.091 0.50
0 98.4 0.13 1.47
0.016 97.6 0.40 1.95
D
OCT3/4 Nanog REX1 SOX2 DNMT3B
Expression Relative to H9 (hESC)
CTRL1 CTRL2 NIPBL1 NIPBL2 NIPBL3 NIPBL4
E
NIPBL1 NIPBL2 NIPBL3 NIPBL4
F
Ectoderm Endoderm Mesoderm
NIPBL1 NIPBL2 NIPBL3 NIPBL4

Supplementary Figure 1. Characterization of iPS cell line from CdLS patient samples.

(A) Chromatographs of heterozygous *NIPBL* mutations (2969delG, 2479_2820delAG, 3503_2A>C, 2479_2480delAG; NIPBL1, NIPBL2, NIPBL3, NIPBL4, respectively) in patient samples. (B) NIPBL gene expression in CTRL- and $NIPBL^{+/-}$-iPSCs, with levels significantly reduced with mutations (*$p < 0.05$, each represents 3 technical replicates analyzed/line). (C) Expression of pluripotency extracellular (SSEA3/4) and intracellular (OCT3/4 and Nanog) antigens. (D) Molecular profile of WT and CdLS iPSC lines. RT-qPCR analysis shows the expression of common pluripotency genes relative to human embryonic stem cell (h9): *OCT3/4, NANOG, REX1, SOX2,* and *DNMT3B.* (E) Representative karyotype analysis of 4 $NIPBL^{+/-}$ iPSC clones showing a normal diploid G-band analysis. (F) Generation of all 3 germ layers in teratoma assay.

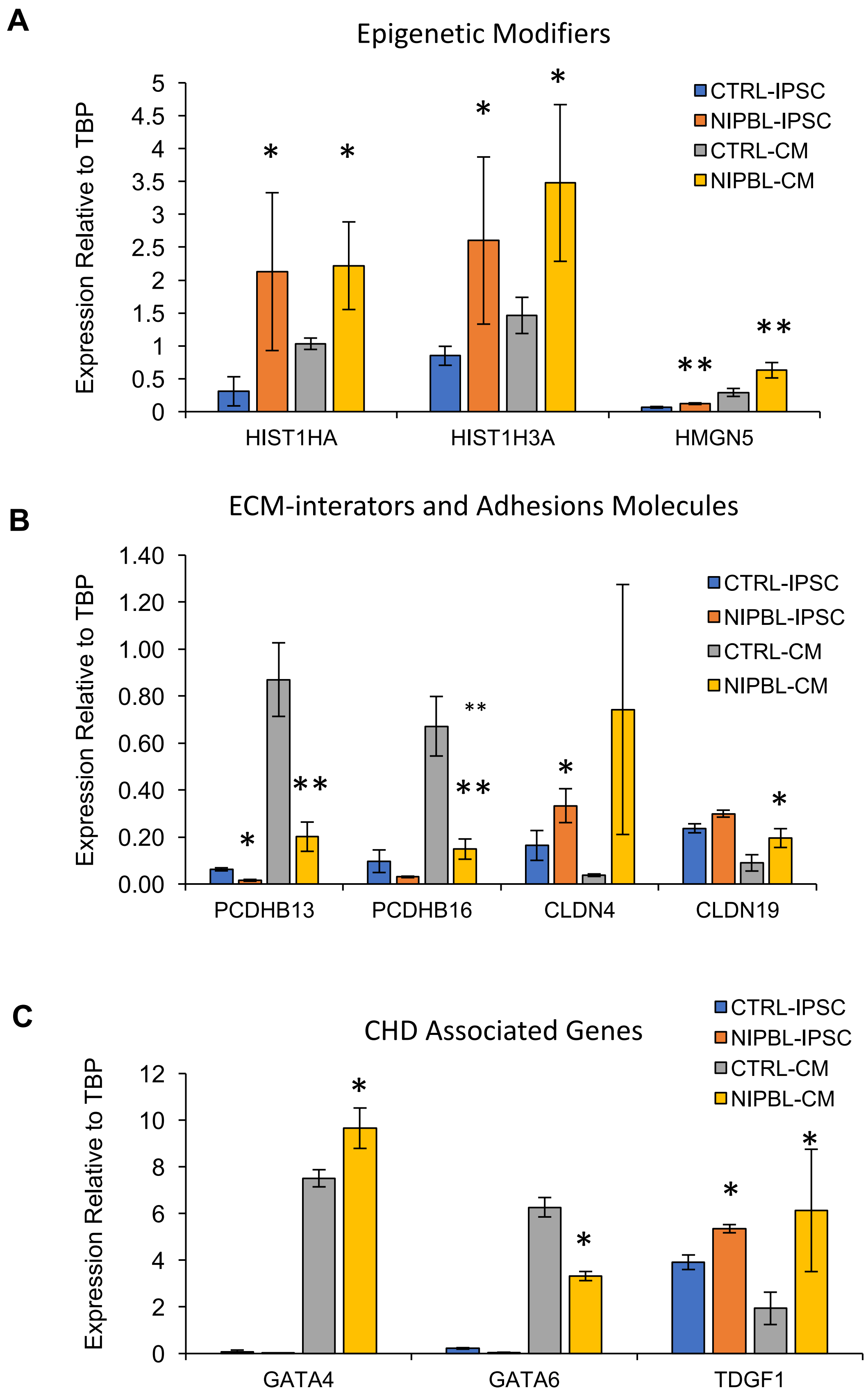
A
Epigenetic Modifiers
Expression Relative to TBP
0
0.5
1
1.5
2
2.5
3
3.5
4
4.5
5
HIST1HA
HIST1H3A
HMGN5
CTRL-IPSC
NIPBL-IPSC
CTRL-CM
NIPBL-CM
B
ECM-interators and Adhesions Molecules
Expression Relative to TBP
0.00
0.20
0.40
0.60
0.80
1.00
1.20
1.40
PCDHB13
PCDHB16
CLDN4
CLDN19
CTRL-IPSC
NIPBL-IPSC
CTRL-CM
NIPBL-CM
C
CHD Associated Genes
Expression Relative to TBP
0
2
4
6
8
10
12
GATA4
GATA6
TDGF1
CTRL-IPSC
NIPBL-IPSC
CTRL-CM
NIPBL-CM

Supplementary Figure 2. Validation of RNASeq data by quantitative RT-PCR.

(A) qRT-PCR in CTRL and NIPBL$^{+/-}$ cells showing major transcriptional activation of epigenetic modifier genes (HIST1H1A, HIST1H3A, and HMGN5) in both iPSCs and CMs. (B) Protocadherin and claudin gene expression is significantly altered in *NIPBL*$^{+/-}$ samples, which are associated with ECM and adhesion molecule gene sets. (C) CHD-associated genes show dysregulated gene expression in NIPBL$^{+/-}$ CMs. Statistics were performed using Student's TTest (*$p < 0.05$). Means were generated from an average of unaffected controls (minimum of n=3) or NIPBL$^{+/-}$ (minimum of n=3) for iPSC or CMs. Error bars are representative of ±SEM.

Supplemental Table 2. PCR primers

GENE	Catalog no.	Company	Label	Orientation	Primer sequence
NIPBL	Hs00209846_m1	Invitrogen	FAM		
TBP	4325803	ABI	VIC		
HIST1H1A	Hs.PT.58.25973099.g	IDT	FAM		
HIST1H3A	Hs.PT.58.88772	IDT	FAM		
HMGN5	Hs.PT.58.3803566	IDT	FAM		
PCDHB13	Hs.PT.58.39662405.g	IDT	FAM		
PCDHB16	Hs.PT.58.26943655.g	IDT	FAM		
CLDN4	Hs.PT.58.1326185.g	IDT	FAM		
CLDN19		IDT		Forward	CAT GGT CCT CAG CGT AGT T
				Reverse	ATA CCT GCC AGG ATG AAG AG
			FAM	Probe	CA TGA AGT GT ACG CGG GTG GGA

GATA6		IDT		Forward	AGACTTGCTCTGGTAATAGCAATA
				Reverse	GAGGCTGTAGGTTGTGTTGT
		IDT	FAM	Probe	TTCCCATGACTCCAACTTCCACCT
GATA4				Forward	GAGATGCGTCCCATCAAGAC
				Reverse	ACATCGCACTGACTGAGAAC
			FAM	Probe	CTGCTGTGCCCGTAGTGAGATGAC
TDGF1	Hs.PT.58.39131775	IDT	FAM		
